# Gamma camera imaging characteristics of ^203/212^Pb as a theragnostic pair for targeted alpha therapy: a feasibility study

**DOI:** 10.1186/s40658-025-00763-2

**Published:** 2025-05-27

**Authors:** David Kästner, Holger Hartmann, Robert Freudenberg, Marc Pretze, Claudia Brogsitter, Michael K. Schultz, Jörg Kotzerke, Enrico Michler

**Affiliations:** 1 Department of Nuclear Medicine, University Hospital Carl Gustav Carus, Technische Universität Dresden, Fetscherstraße 74, 01307 Dresden, Germany; 2 Perspective Therapeutics Inc, Coralville, IA USA; 3 Faculty of Medicine Carl Gustav Carus, Technische Universität Dresden, Dresden, Germany

**Keywords:** Pb-212, Pb-203, SPECT/CT, Gamma camera, Theragnostics, Targeted alpha therapy, Radionuclide therapy

## Abstract

**Background:**

^203^Pb and ^212^Pb show promise as theragnostic agents for targeted alpha therapy (TAT) because two chemically identical isotopes can be used for diagnostic imaging and treatment. In the ^212^Pb decay chain, in addition to alpha and beta particles, a large number of photons are emitted, those with an energy of 239 keV and the characteristic X-rays of ^212^Pb could be used for imaging. ^203^Pb decays by photon emission with an energy of 279 keV, which appears suitable for gamma camera imaging. The aim of this study was to investigate suitable imaging protocols and to characterize the scintigraphic imaging properties and their implications for the clinical feasibility as theragnostic isotopes.

**Methods:**

Planar and SPECT/CT images were obtained with medium- and high-energy collimators on a Siemens Symbia Intevo 6 using a NEMA image quality phantom in various phantom setups and another body-shaped phantom with several inserts. Different energy windows were investigated and measurements were evaluated in terms of sensitivity, count rate performance, spatial resolution, contrast recovery, lesion detectability, and image quantification.

**Results:**

Evaluation of image quality showed superior imaging characteristics for ^203^Pb compared to ^212^Pb regarding spatial resolution, contrast recovery, image noise, and quantification accuracy. Both medium- and high- energy collimators were suitable for ^203^Pb imaging, with the medium energy collimators showed slightly better imaging properties. Images obtained with the HE collimators in the 79 keV energy window showed the best visual image quality for ^212^Pb. Due to high-energy photon emissions from ^212^Pb daughter nuclides (e.g., 2.6 MeV from ^208^Tl), dead time related count losses occurred even at low activities (20% count loss at 20 MBq for MELP collimators).

**Conclusions:**

According to our results and first-in-human imaging studies, SPECT/CT imaging with the ^203/212^Pb theragnostic pair is clinically feasible. ^203^Pb is an appropriate imaging surrogate to investigate pharmacokinetics and perform predictive dosimetry. The less favorable imaging characteristics of ^212^Pb make image quantification and post-treatment dosimetry challenging and require further research.

## Background

Alpha-particle-emitting radionuclides are becoming increasingly important in radionuclide therapy for cancer. The higher linear energy transfer (LET) and the short path lengths of alpha particles compared to electrons result in an increased incidence of double-strand DNA breaks and increased relative biological effectiveness (RBE) [1–3]. Some of the most studied alpha-particle emitters are radium-223 (^223^Ra) and actinium-225 (^225^Ac), which demonstrate high potential for targeted-alpha-therapy (TAT) of various cancers [4–8]. Planar scintigraphy, SPECT/CT imaging and dosimetry approaches have been investigated for these radionuclides [9–14]. However, imaging of ^225^Ac is challenging in a routine setting due to the low photon emission probability, low administered activity, and resulting long acquisition times. As imaging is a prerequisite for the investigation of pharmacokinetics and derived dosimetry of alpha-particle radiopharmaceuticals, the use of an imaging surrogate allows for PET or SPECT imaging by exchanging the alpha-emitting therapeutic radionuclide with a positron or gamma-emitting radionuclide [15, 16]. In addition, an imaging surrogate can be used to identify patients who may benefit from a TAT and to perform a predictive dosimetry and treatment planning in a theragnostic approach.

**Fig. 1:**
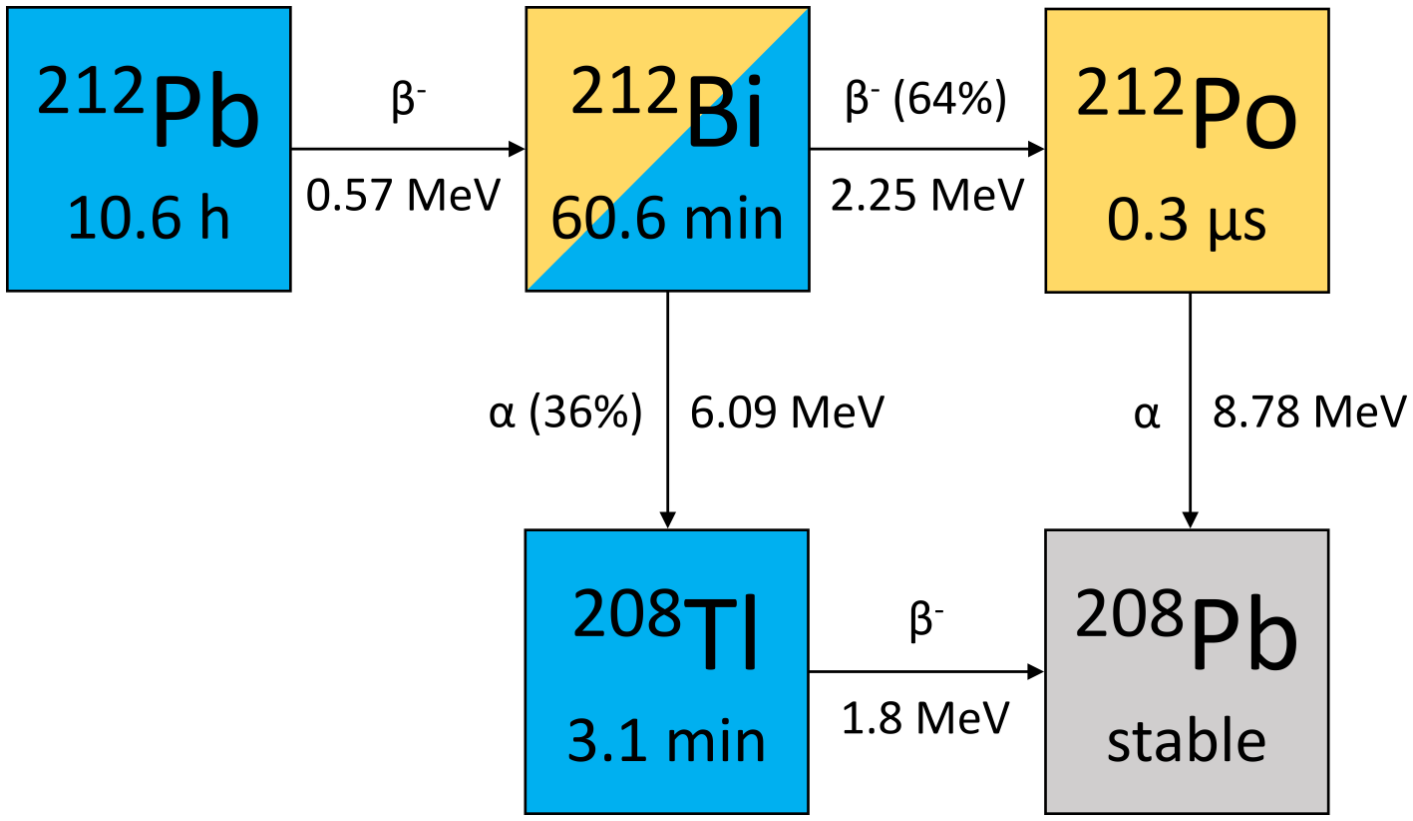
Decay chain of ^212^Pb with maximum electron energies and alpha-particle energies [37, 38]

A new promising radionuclide for TAT is lead-212 (^212^Pb) (t_1/2_=10.6 h), which was first introduced clinically by Meredith et al. [17, 18]. In the decay chain of the beta-emitter ^212^Pb to stable lead-208 (^208^Pb), one α-particle is emitted per decay via bismuth-212 (^212^Bi) or polonium-212 (^212^Po) (Fig. 1). In addition, there are imageable gamma and X-ray emissions. Ongoing preclinical and clinical studies are investigating the potential of novel ^212^Pb-labeled peptides and antibodies [19–28], and recently the first in-human SPECT/CT imaging of ^212^Pb in a patient with a neuroendocrine tumor was presented by our department [29]. An advantage of ^212^Pb is the availability of the elementally matched isotope lead-203 (^203^Pb) as an imaging surrogate. With its high gamma emission probability and longer half-life of 51.9 h, it is considered an ideal theragnostic partner for assessing pharmacokinetics and performing individualized dosimetry. There are some studies investigating ^203^Pb as a surrogate isotope for ^212^Pb and first clinical planar and SPECT/CT imaging have been performed [25, 30–34]. However, further fundamental studies on the scintigraphic imaging properties of ^203^Pb and ^212^Pb are required to exploit the potential of this promising theragnostic pair.

The aim of the present study was to investigate suitable acquisition protocols for scintigraphic imaging of ^203^Pb and ^212^Pb and to characterize and compare the scintigraphic imaging properties of both isotopes. For this purpose, planar and SPECT/CT images of different phantoms were acquired. The use of different energy windows and collimators was investigated and the images were evaluated with regard to sensitivity, count rate performance, spatial resolution, contrast recovery, lesion detectability, and image quantification. In addition, implications of the identified imaging characteristics with respect to clinical feasibility as theragnostic isotopes for pre- and post-treatment imaging and dosimetry are discussed.

## Methods

### Physical properties, production, and activity measurements of ^203^Pb and ^212^Pb

The isotope ^203^Pb (t_1/2_=51.9 h) decays by electron capture to stable thallium-203 (^203^Tl), emitting 279 keV photons suitable for scintigraphic imaging. It can be produced via the ^205^Tl(p,3n)^203^Pb reaction by irradiating enriched ^205^Tl targets with high-energy protons [35]. The ^203^Pb used for the measurements in this study was obtained from the Medical Isotope and Cyclotron Facility at the University of Alberta (Edmonton, AB, Canada). The beta-emitter ^212^Pb decays with a half-life of 10.6 h via the alpha-emitters ^212^Bi and ^212^Po ultimately to stable ^208^Pb (Fig. 1). In addition to alpha- and beta-emissions, several gammas and characteristic X-rays are emitted by ^212^Pb and its progeny (Table 1). ^212^Pb can be produced from radium-224 (^224^Ra) using an isotope generator. For this study, a ^224^Ra/^212^Pb generator (VMT-α-GEN) manufactured by Perspective Therapeutics, Inc. (Coralville, IA, USA) was used. Radionuclide purity of both isotopes was assessed by gamma spectroscopy using a high purity germanium (HPGe) detector (Canberra Industries, Meriden, CT, USA). Radioactivity measurements for the experiments in this study were performed using a dose calibrator (ISOMED 2010, NuviaTech Healthcare, Germany). The dose calibrator was calibrated using a reference solution of ^203^Pb or ^212^Pb and the calibration was then verified by HPGe detector measurements.

### SPECT/CT camera characteristics and energy window definition

All images were acquired using a dual-head Symbia Intevo 6 SPECT/CT scanner (Siemens Healthineers, Erlangen, Germany) with 3/8 inch NaI(Tl) crystals. The rectangular field of view (FOV) was 53.5 cm x 38,7 cm. Two types of collimators, medium-energy low-penetration (MELP) collimators (hole length: 40.64 mm, septal thickness: 1.14 mm, hole diameter: 2.94 mm) and high-energy (HE) collimators (hole length: 59.7 mm, septal thickness: 2.0 mm, hole diameter: 4.0 mm), were evaluated for both isotopes. The use of low-energy collimators was considered inappropriate due to the relatively high photon energies of both isotopes and the high-energy emissions of the ^212^Pb progeny. The energy windows were selected based on the emission data (Table 1) and the energy spectra of ^203^Pb and ^212^Pb. For ^203^Pb, a photopeak window was defined at 279 keV (20% width) with adjacent upper and lower scatter windows of 10% at 237 keV and 321 keV. An additional energy window for the detection of ^203^Pb characteristic X-rays was set to 72 keV (40% width) with adjacent scatter windows of 20% at 50 keV and 94 keV. For ^212^Pb, the energy windows were defined according to Kvassheim et al. [36]. The ^212^Pb photopeak window was centered at 239 keV (20% width) with two adjacent scatter windows of 5% at 209 keV and 269 keV. Another energy window for the ^212^Pb characteristic X-rays was set to 79 keV (40% width) with upper and lower scatter windows of 20% centered at 55 keV and 103 keV. The percentage width of the scatter windows was defined relative to the respective photopeak window center.

**Table 1:** Gamma and X-ray emission data for ^203^Pb and ^212^Pb and its daughter nuclides ^212^Bi, ^212^Po and ^208^Tl. Photon energies with emission probabilities greater than 1% are included [37–39]

Nuclide	Daughter nuclides (unstable)	Gamma energies (keV) & emission probabilities (%)	X-ray energies (keV) & emission probabilities (%)
^203^Pb		279.2 (80.9) 401.3 (3.4)	70.8 (25.5) 72.9 (43.0) 82.1–85.1 (19.0)
^212^Pb		238.6 (43.6) 300.1 (3.3)	74.8 (10.0) 77.1 (16.8) 86.8–90.1 (7.5)
	^212^Bi	727.3 (6.7) 785.4 (1.1) 1620.5 (1.5)	-
	^212^Po	-	-
	^208^Tl	277.4 (6.6) 510.8 (22.6) 583.2 (85.0) 763.1 (1.8) 860.6 (12.5) 2614.5 (99.8)	72.8 (2.1) 75.0 (3.5) 84.5–87.6 (1.5)

### Sensitivity and count rate performance

Sensitivity measurements were performed using a fillable line source (length 18 cm) placed on the patient bed in the center of the FOV without attenuating material with activities of 10.3 MBq and 9.9 MBq for ^203^Pb and ^212^Pb, respectively. Planar images were acquired for 5 min on a 256 × 256 matrix with MELP and HE collimators. The source-detector distance was set to 10 cm. Sensitivities were calculated for each energy window as the total number of counts in the FOV divided by the acquisition time and the measured activity.

**Fig. 2:**
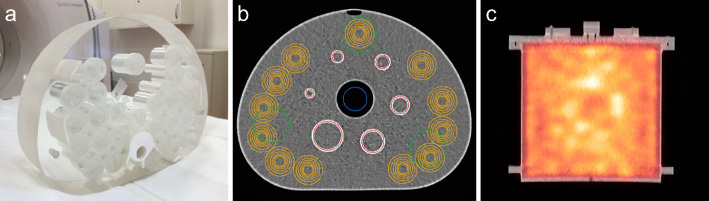
**(a)** Custom-made phantom with a body-shaped cross section, including parts of the ribs and spine used for count rate performance measurements. **(b)** CT image of the NEMA image quality phantom. Transverse slice at the level of the spheres. Sphere VOIs (red), 12 corresponding spherical background VOIs (orange), 3 large cylindrical background VOIs (green), lung insert VOI (blue). **(c)** SPECT/CT fusion image (coronal slice) of the uniform cylindrical phantom used for determination of calibration factors

The count rate linearity and dead time effects were analyzed as a function of the amount of activity measured. An activity-filled vial was placed in a hole at the center of a custom-made phantom. The phantom is made of polymethylmethacrylate (PMMA) and has a body-shaped cross section (diameter 30 cm, width 8 cm), including parts of the ribs and spine (made of polytetrafluorethylene (PTFE)) with bone-equivalent tissue (Fig. 2a) to simulate patient attenuation and scattering conditions. Activities ranged from 18 MBq to 1659 MBq for ^203^Pb and 5 MBq to 290 MBq for ^212^Pb. Planar images were acquired for 2 min on a 256 × 256 matrix. Anterior and posterior detector-phantom center distances were set to 22 cm and 17 cm, respectively. For data analysis, the counts in the photopeak window were plotted against the activity in the phantom. For each isotope, energy window and collimator, a data fit was performed according to the paralyzable detector model (PDM) as follows:

1 $$\:C\left(A\right)=\alpha\:\cdot\:A\cdot\:{e}^{-\tau\:\cdot\:\alpha\:\cdot\:A}$$

where *C* is the measured count rate, *A* is the activity at scan time, *α* describes the linear detector performance and *τ* is the detector dead time. Data points where the measured count rate decreased were not fitted. The activities at which the count rate begins to decrease according to the PDM fit were determined and activities at 10% and 20% count rate loss were calculated. In addition, energy spectra were measured and analyzed for each acquisition.

### Quantitative and qualitative evaluation of image quality

#### Phantom setups

To assess the quantitative and qualitative image quality, a torso-shaped NEMA image quality (IQ) phantom was used, comprising a fillable background compartment, six fillable coplanar spheres with inner diameters of 10, 13, 17, 22, 28, and 37 mm respectively, and a cylindrical lung insert. The phantom was filled with three different sphere-to-background activity concentration ratios to resemble clinical setups. To emulate bone metastases, which are characterized by activity spots with low background activity, the phantom was measured with activity in the six spheres only. To simulate liver metastases with different tumor-to-background ratios, the spheres and the background compartment were filled. Sphere-to-background activity concentration ratios of 8:1 and of 4:1 were used. The phantom was filled with ^203^Pb or ^212^Pb with an activity concentration of approximately 40 kBq/ml in the spheres. The activity concentration in the background compartment was approximately 5 kBq/ml or 10 kBq/ml. The actual activity concentrations, sphere-to-background activity concentration ratios, and the total activity within the phantom at the time of imaging are shown in Table 2.

**Table 2:** Information on the measurements with the NEMA image quality phantom filled with ^203^Pb and ^212^Pb in different Phantom setups (spheres without background activity; sphere-to-background activity concentration ratios 8:1 and 4:1)

Nuclide	Phantom setup	Activity concentration in spheres (kBq/ml)	Activity concentration in background (kBq/ml)	Sphere-to-background activity concentration ratio	Total activity in the phantom at scanning time (MBq)
^203^Pb	Spheres without background	39.8	-	-	1.78
	Contrast 8:1	38.4	4.8	7.73:1	46.94
	Contrast 4:1	37.3	9.3	3.94:1	89.38
^212^Pb	Spheres without background	39.1	-	-	1.75
	Contrast 8:1	31.6	4.0	7.71:1	38.67
	Contrast 4:1	25.8	6.4	3.98:1	61.04

#### Planar imaging of the NEMA IQ Phantom

Planar anterior and posterior images were acquired for each phantom setup to evaluate the planar scintigraphic imaging characteristics of ^203^Pb and ^212^Pb. Imaging was performed with an acquisition time of 5 min on a 256 × 256 matrix for each collimator and for the photopeak and the characteristic X-ray energy windows. The phantom was rotated and positioned in such a way that all the spheres could be imaged. The image quality was visually evaluated.

#### SPECT/CT acquisition and image reconstruction

All SPECT data was acquired over 360° with 120 projections (60 per detector head, 30 s per projection) on a 256 × 256 matrix using a body contouring orbit and step-and-shoot mode. The SPECT scan was followed by a low-dose CT scan (130 kV, 20 mAs, 2.5 mm slice thickness), which was used for attenuation correction. Images were reconstructed to a voxel size of 2.4 × 2.4 × 2.4 mm^3^ using the 3D OSEM algorithm (Flash 3D; Siemens Healthineers) with 16 iterations and 8 subsets. Post-reconstruction Gaussian filtering of 9 mm and 12 mm was applied for ^203^Pb and ^212^Pb, respectively. The larger Gaussian filter was chosen for ^212^Pb due to the lower gamma emission probability and therefore lower count statistics and expected higher image noise. The number of iterations were chosen according to the results of Kvassheim et al. [36]. Scatter correction was performed using the triple energy window method [40] to correct for down-scattered high-energy photons. The acquisition times were adjusted over time according to the respective half-life.

#### Image analysis

The reconstructed SPECT images were evaluated in terms of contrast recovery, image noise, lesion detectability, relative count error in the lung insert, and spatial resolution. The six hot spheres were segmented using spherical volumes of interest (VOI) with a diameter equal to the inner diameter of each sphere. The VOIs were centered using the CT. For the background, 12 spherical VOIs (BG) with a diameter equal to the spheres were defined for each sphere, resulting in a total of 72 background regions. Additionally, three cylindrical VOIs (45 mm diameter, 150 mm length) were drawn to evaluate a large volume of the phantom background. The lung insert was delineated using a cylindrical centered VOI (30 mm diameter, 130 mm length) (Fig. 2b). The contrast recovery coefficient (CRC) was calculated for each of the six spheres according to the NEMA NU 2-2018 protocol [41] as the correlation between the measured and true sphere-to-background activity concentration ratio. The relative count error in the lung insert (ΔN_Lung_) was determined as the ratio of the mean number of counts in the lung insert VOI to the mean number of counts in the three large background VOIs. To evaluate the image noise, the background coefficient of variation (CV_BG_) was calculated using the standard deviation of all voxels within the three background VOIs divided by the mean number of counts in these background VOIs. The contrast-to-noise ratio (CNR) was used to assess object detectability and was calculated for each sphere as follows:

2 $$\:CNR=\frac{{\stackrel{-}{N}}_{\text{S}}-{\stackrel{-}{N}}_{\text{BG}}}{{\sigma\:}_{\text{BG}}}$$

where $\:{\stackrel{-}{N}}_{\text{S}}$ is the mean number of counts in the sphere VOI, $\:{\stackrel{-}{N}}_{\text{BG}}$ is the mean number of counts in the corresponding 12 background VOIs, and $\:{\sigma\:}_{\text{BG}}$ is the standard deviation of the counts in the 12 background VOIs. The tomographic spatial resolution was determined based on the analysis of radial profiles through the homogeneously filled spheres in the reconstructed images according to [42]. The full width at half maximum (FWHM) of the point spread function was assessed using the software Rover (version 3.0.6.74 h, ABX, Germany).

#### Quantitative imaging

To perform SPECT quantification and convert counts per voxel to Bq/ml, calibration factors (CFs) were determined using a uniform cylindrical phantom with a volume of 5650 ml (Fig. 2c). The phantom was filled with activity concentrations ($\:\stackrel{-}{A})$ of 17.6 kBq/ml (total activity 99.4 MBq) and 2.56 kBq/ml (total activity 14.6 MBq) for ^203^Pb and ^212^Pb, respectively. A large VOI was placed in the reconstructed cylinder volume to obtain the total number of counts within the VOI (*N*_*VOI*_). The calibration factor was determined as:

3 $$\:CF=\frac{{N}_{VOI}}{{V}_{VOI}\cdot\:t\:\cdot\:\stackrel{-}{A}}$$

where *t* is the acquisition time and *V*_*VOI*_ is the volume of the VOI. Acquisition and reconstruction were performed as described previously for the other phantom measurements.

To assess the quantification accuracy, recovery coefficients (RCs) were calculated for each of the six spheres and the background compartment in the NEMA IQ phantom. The recovery coefficient was computed as:

4 $$\:RC=\frac{{\stackrel{-}{A}}_{SPECT}}{{\stackrel{-}{A}}_{true}}$$

where $\:{\stackrel{-}{A}}_{SPECT}$ is the measured activity concentration and $\:{\stackrel{-}{A}}_{true}$ is the known activity concentration in the spheres or in the background compartment. The activity concentration in the background compartment was determined using the three cylindrical VOIs.

## Results

### Sensitivity and count rate performance

The sensitivities measured for ^203^Pb and ^212^Pb for the photopeak and the characteristic X-ray windows and for the MELP and HE collimators are listed in Table 3. For ^203^Pb, the measurements with the 72 keV characteristic X-ray energy window showed a three times higher sensitivity than the measurements with the 279 keV photopeak window. For ^212^Pb, the measurements with the 79 keV characteristic X-ray energy window showed a slightly lower sensitivity compared to the measurements with the 239 keV photopeak window. For both isotopes, the MELP collimators showed a higher sensitivity than the HE collimators.

**Fig. 3:**
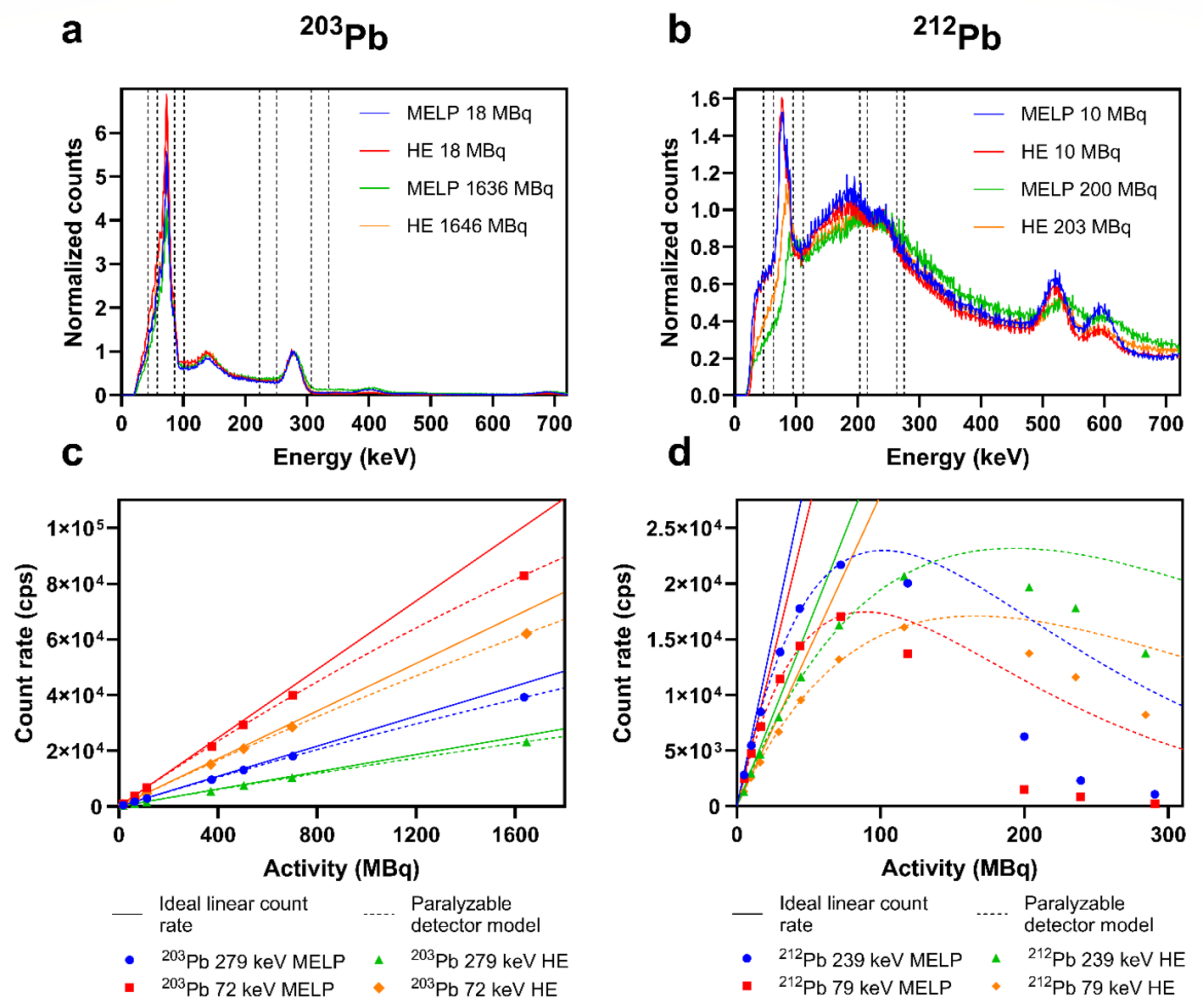
Energy spectra of ^203^Pb **(a)** and ^212^Pb **(b)** at low and high activities obtained on the SPECT/CT scanner with MELP and HE collimators. The curves are normalized to the peak maximum at 279 keV for ^203^Pb and 239 keV for ^212^Pb. Energy windows according to the TEW method are shown by the dashed lines. Count rate performance measured for ^203^Pb **(c)** and ^212^Pb **(d)** with MELP and HE collimators

Figure 3a-b show the energy spectra of ^203^Pb and ^212^Pb acquired with MELP and HE collimators at low and high activities. In the ^203^Pb spectra, the photopeaks at 279 keV and 401 keV and the characteristic X-rays were visible. The characteristic X-ray peak was lower for the measurements at high activities compared to the measurements at low activities. In the ^212^Pb spectra, the photopeak at 239 keV and the characteristic X-rays were visible. In addition, the ^208^Tl photopeak at 583 keV and a peak at 511 keV could be observed. The spectra obtained at high activities show peak broadening and peak shifting and a decrease of the X-ray peak relative to the photopeak at 239 keV. In Fig. 3c-d, the count rate response for ^203^Pb and ^212^Pb measured with MELP and HE collimators in different energy windows are shown. For ^203^Pb, there was a moderately increasing deviation from the ideal count rate with increasing activity for all measurements. The lowest activity with a 10% count loss was 899 MBq measured with the 72 keV window and the MELP collimators (Table 3). For ^212^Pb, a progressive count rate loss could be observed with increasing activity. The activities measured for a 20% count rate loss were very low between 20 MBq and 43 MBq (Table 3). The activities where the count rates according to the PDM decreased were 102 MBq and 89 MBq for the MELP collimators and 193 MBq and 166 MBq for the HE collimators, measured with the 239 keV and 79 keV energy window, respectively. For both isotopes, higher count rate losses occurred for the characteristic X-ray window compared to the photopeak window and for the MELP collimators compared to the HE collimators.

**Table 3:** Sensitivities and estimated activities at 10% and 20% count rate loss for the planar measurements with ^203^Pb and ^212^ Pb

Nuclide	^203^Pb				^212^Pb			
**Collimator**	MELP		HE		MELP		HE	
**Energy window**	279 keV	72 keV	279 keV	72 keV	239 keV	79 keV	239 keV	79 keV
**Sensitivity (cps/MBq)**	78.8	242.0	45.2	173.9	1148.9	965.2	622.7	524.3
**A (MBq) at 10% count loss**	1425	899	1863	1391	11	9	20	18
**A (MBq) at 20% count loss**	3017	1906	3945	2946	23	20	43	37

### Quantitative and qualitative evaluation of image quality

#### Planar imaging of the NEMA IQ phantom

**Fig. 4:**
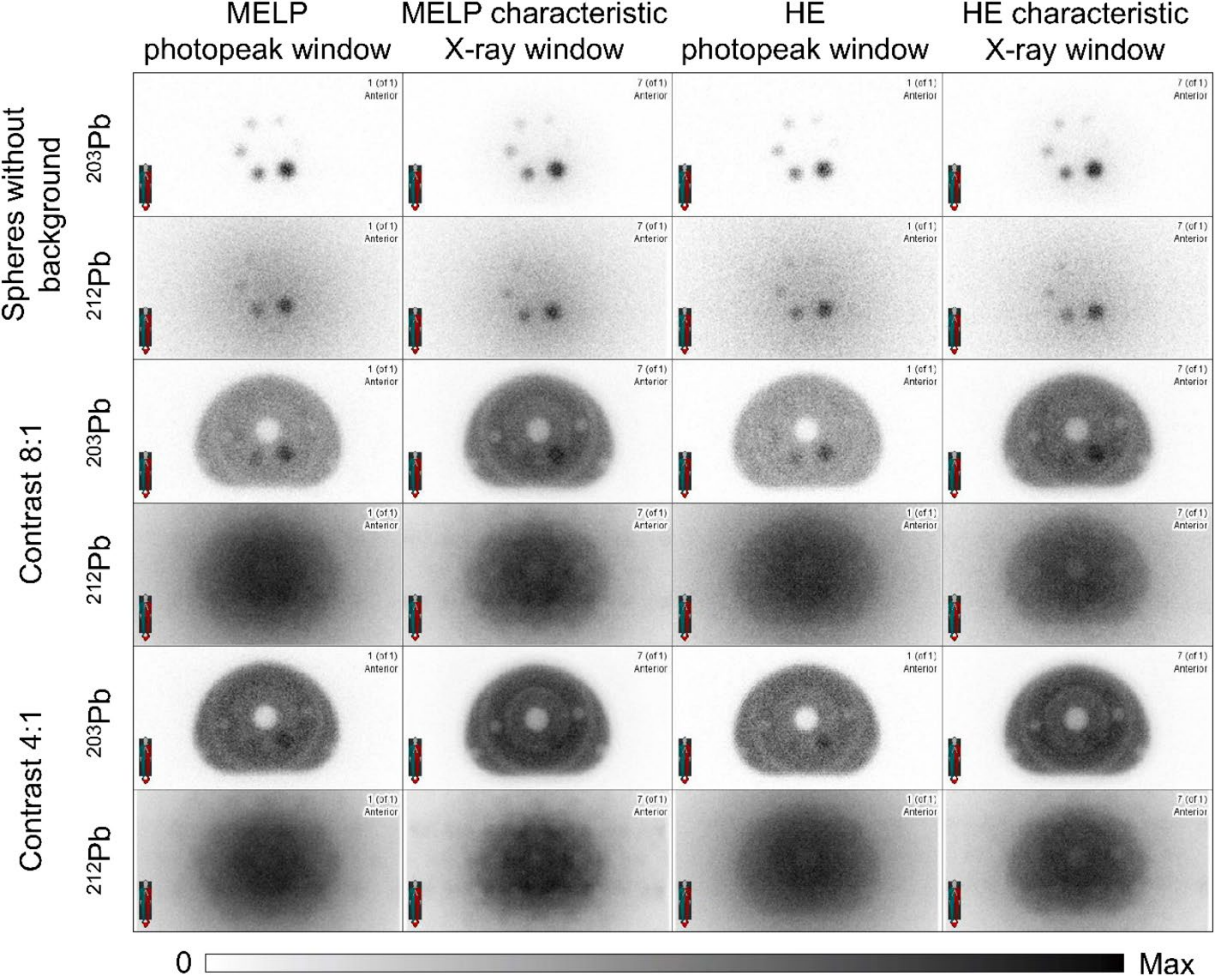
Planar anterior gamma camera images of the NEMA image quality phantom filled with ^203^Pb and ^212^Pb in different phantom setups (spheres without background activity, sphere-to-background activity concentration ratio 8:1 & 4:1) acquired with MELP and HE collimators and different energy windows (photopeak window: 279 keV (^203^Pb) & 239 keV (^212^Pb); characteristic X-ray window: 72 keV (^203^Pb) & 79 keV (^212^Pb))

Figure 4 shows the planar images of the NEMA IQ phantom in all phantom setups of ^203^Pb and ^212^Pb. For ^203^Pb, the images acquired with the 279 keV energy window showed a better detectability of the spheres and lower artificial counts in the activity-free lung insert than the images acquired with the characteristic X-ray window. For the acquisitions of ^203^Pb without background activity, all spheres except the smallest sphere (d = 10 mm) were visible. In the 8:1 contrast images only two spheres (d ≥ 28 mm) and in the 4:1 contrast images only the largest sphere (d = 37 mm) were detectable. In the ^212^Pb images with activity only in the spheres, the four largest spheres (d ≥ 17 mm) were detectable. For the acquisitions with background activity, only the shape of the phantom was visible and none of the spheres were detectable. The lung insert could only be distinguished from the background in the images acquired with the HE collimators and the 79 keV energy window. All acquisitions of ^212^Pb showed a high image and background noise. The ^212^Pb images with activity in the background compartment showed artefacts related to image non-uniformity and the photomultiplier tube (PMT) outlines were visible.

#### SPECT/CT imaging of ^203^Pb with different phantom setups

**Fig. 5:**
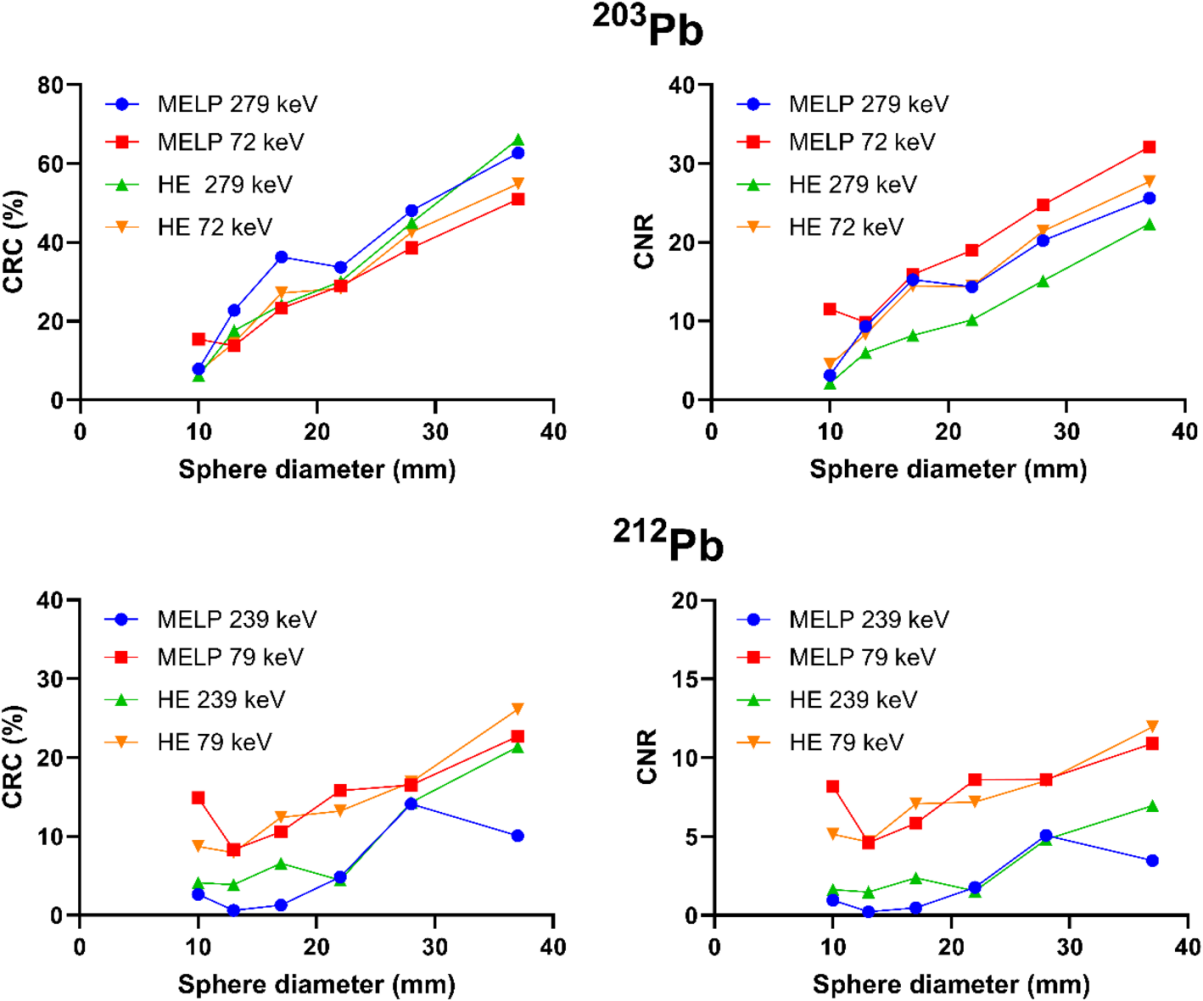
Contrast recovery coefficients (CRC) and contrast-to-noise ratios (CNR) as a function of sphere diameter for ^203^Pb (upper row) and ^212^Pb (lower row) determined using the NEMA image quality phantom at a sphere-to-background activity concentration ratio of 8:1 acquired with MELP and HE collimators and different energy windows

Figure 5 depicts the CRC and the CNR as a function of sphere diameter for the NEMA IQ phantom measurements of ^203^Pb with a sphere-to-background activity concentration ratio of 8:1. For the images acquired with the 279 keV energy window, the CRCs were slightly higher compared to the 72 keV window images. The CRCs of the largest sphere measured with the MELP collimators were 62.7 and 51.0 for the 279 keV and 72 keV window, respectively. In contrast, the CNRs were slightly lower for the 279 keV window images compared to 72 keV window images. Spatial resolution, lung count error, and image noise determined for the different phantom setups are listed in Table 4. For the 279 keV energy window measurements, the spatial resolution was approximately 0.5 mm better than for the 72 keV window measurements. The MELP collimator images showed an up to 0.9 mm better resolution compared to the HE collimator. The spatial resolution was worse for the images with background activity compared to the images with activity in the spheres only. The lung count error was up to twice as high for the 72 keV window compared to the 279 keV window measurements. The image noise was slightly worse for the HE collimators compared to the MELP collimators (noise level 19.6% vs. 17.7%).

**Fig. 6:**
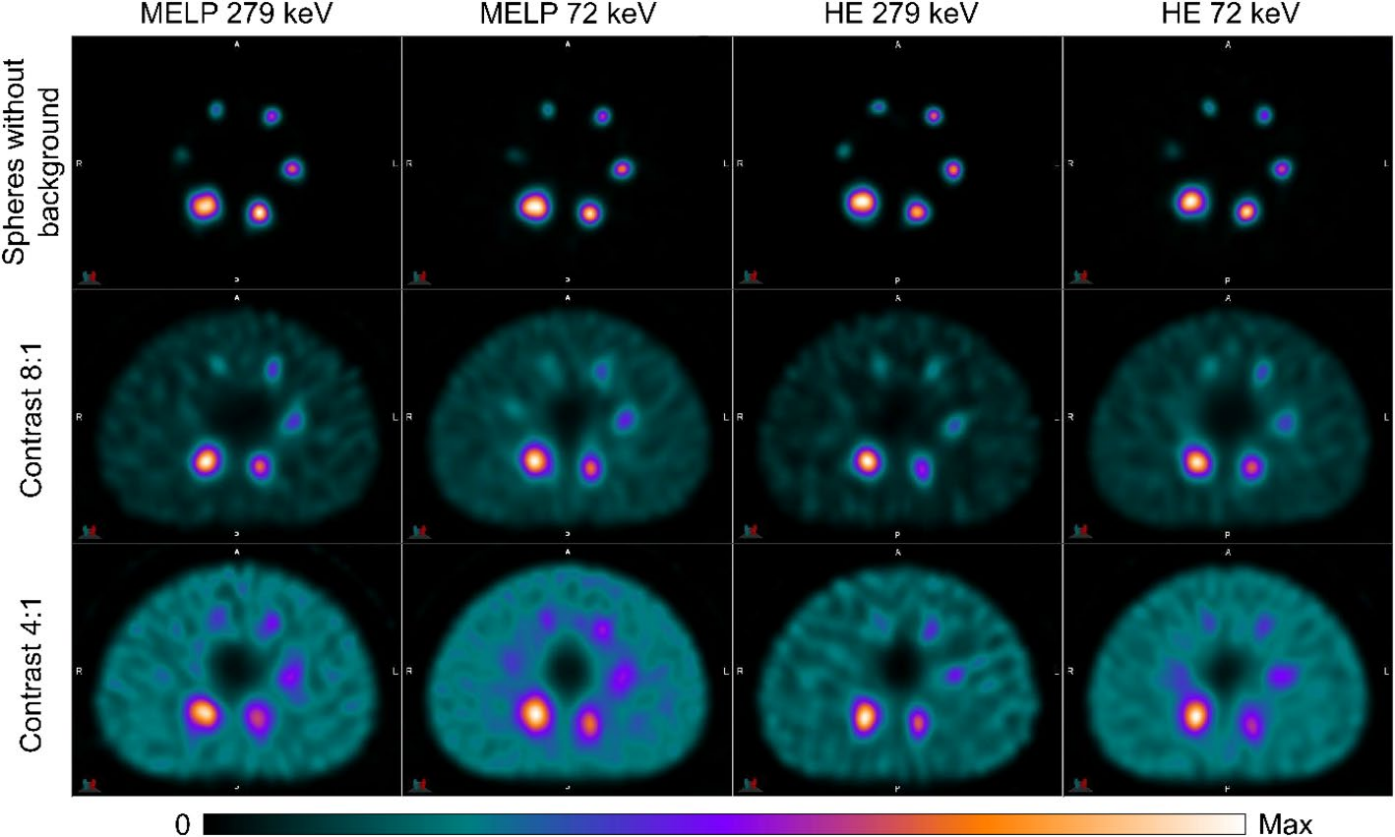
SPECT images of the NEMA image quality phantom filled with ^203^Pb in different phantom setups (spheres without background activity, sphere-to-background activity concentration ratio 8:1 & 4:1). Images were acquired with MELP and HE collimators using energy windows at 279 keV and 72 keV. Transverse slices at the level of the spheres are shown

Figure 6 shows the reconstructed transverse images of all NEMA IQ phantom setups of ^203^Pb acquired with MELP and HE collimators and different energy windows. All spheres were visible for the measurements with activity in the spheres only. For the images with a sphere-to-background contrasts of 8:1 and 4:1, the smallest sphere (d = 10 mm) was not detectable, except for the images acquired with MELP collimators in the 72 keV window, where all spheres were detectable. Higher image noise was observed in the images acquired in the 279 keV energy window compared to the 72 keV window.

**Table 4:** Spatial resolution (FWHM), image noise (CV_BG_), lung count error (ΔN_Lung_), and background recovery (RC_BG_) determined using the NEMA image quality phantom filled with ^203^Pb and ^212^Pb in different phantom setups (spheres without background activity, contrast 8:1 and 4:1)

Phantom setup	Nuclide	Collimator	Energy window	FWHM (mm)	CV_BG_ (%)	ΔN_Lung_ (%)	RC_BG_
Spheres without background	^203^Pb	MELP	279 keV	12.2	-	-	-
			72 keV	12.7	-	-	-
		HE	279 keV	13.1	-	-	-
			72 keV	13.4	-	-	-
	^212^Pb	MELP	239 keV	14.8	-	-	-
			79 keV	14.9	-	-	-
		HE	239 keV	15.2	-	-	-
			79 keV	14.8	-	-	
Contrast 8:1	^203^Pb	MELP	279 keV	14.5	17.65	24.05	1.05
			72 keV	14.9	12.33	38.22	1.01
		HE	279 keV	15.1	19.64	17.55	1.02
			72 keV	15.5	13.8	33.62	1.01
	^212^Pb	MELP	239 keV	18.7	21.77	67.87	0.78
			79 keV	17.1	14.94	88.58	0.87
		HE	239 keV	19.4	22.08	81.25	0.81
			79 keV	16.9	15.26	79.83	0.92
Contrast 4:1	^203^Pb	MELP	279 keV	15.7	12.27	21.96	1.02
			72 keV	16.4	10.52	38.01	0.99
		HE	279 keV	16.2	14.84	14.5	0.98
			72 keV	16.6	11.44	34.43	0.99
	^212^Pb	MELP	239 keV	31.9	20.57	70.6	0.46
			79 keV	n.a.	16.42	82.06	0.61
		HE	239 keV	35.4	18.39	75.06	0.59
			79 keV	25.1	14.27	82.96	0.81

#### SPECT/CT imaging of ^212^Pb with different phantom setups

Figure 5 shows the CRC and the CNR as a function of sphere diameter for the NEMA IQ phantom measurements of ^212^Pb with a contrast ratio of 8:1. The CRCs and the CNRs were higher for the 79 keV energy window measurements compared to the 239 keV window measurements. Spatial resolution, lung count error, and image noise determined for the different phantom setups are listed in Table 4. The spatial resolution was up to 20 mm worse for the images with 4:1 contrast compared to the images with 8:1 contrast and spheres without background activity. For the 4:1 contrast acquisition with the HE collimators in the 79 keV window, the spatial resolution could not be determined because the FHWM fit did not converge. The measured count errors in the lung insert were relatively high, exceeding 70% for almost all acquisitions. The noise level was comparable between the MELP and HE collimators, but considerably lower for the 79 keV window compared to the 239 keV window (14.9% vs. 21.8%).

**Fig. 7:**
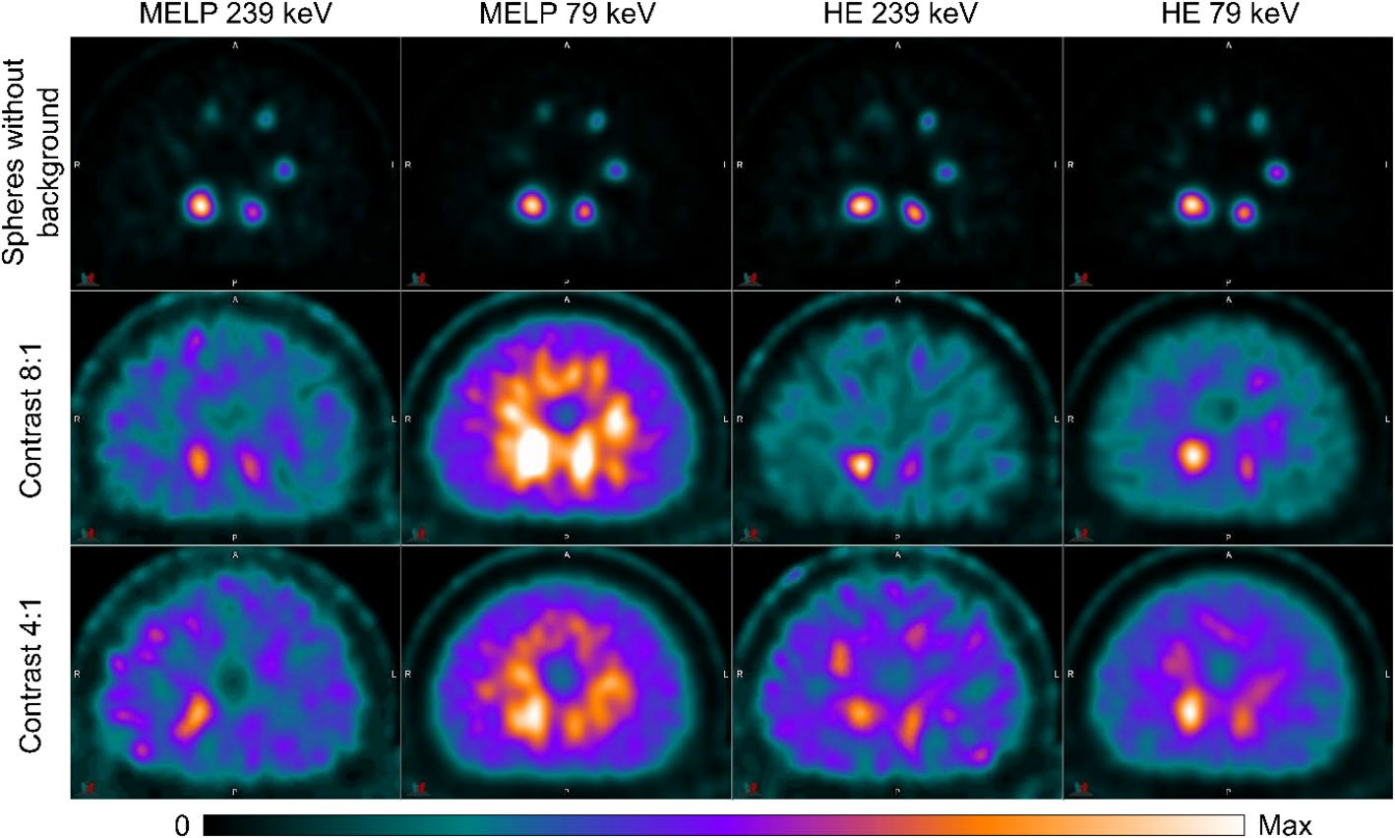
SPECT images of the NEMA image quality phantom filled with ^212^Pb in different phantom setups (spheres without background activity, sphere-to-background activity concentration ratio 8:1 & 4:1). Images were acquired with MELP and HE collimators using energy windows at 239 keV and 79 keV. Transverse slices at the level of the spheres are shown

The reconstructed transverse images of all NEMA IQ phantom setups and all acquisition protocols of ^212^Pb are shown in Fig. 7. For the acquisitions with activity only in the spheres, all spheres except the smallest sphere (d = 10 mm) were clearly visible. In the 8:1 and 4:1 contrast images, it was difficult to distinguish the spheres from the noisy background. The images obtained with the HE collimators in the 79 keV window showed the best visibility of the spheres, with four and two of the six spheres detectable in the 8:1 and 4:1 contrast images, respectively. There was a higher image noise and a blurring at the edge of the phantom in the images obtained in the 239 keV energy window compared to the 79 keV window. The images with activity in the background showed a distortion of the spheres and orbit artefacts.

#### Quantitative imaging

The calculated calibration factors for all acquisition protocols of ^203^Pb and ^212^Pb are presented in Table 5. The CFs were higher for the MELP collimators compared to the HE collimators and additionally higher for the characteristic X-ray energy windows compared to the photopeak windows. The CFs determined for the HE collimators and the characteristic X-ray energy windows are almost identical for ^203^Pb and ^212^Pb, although the planar sensitivities (Table 3) were different. In addition, the CFs for ^212^Pb for the 79 keV window were up to four times higher than the CFs for the 239 keV window, despite slightly lower planar sensitivities for the 79 keV window. In Table 4, the background recovery coefficients are shown. For ^203^Pb, the deviations of the measured activity concentration from the true activity concentration in the background were less than or equal to 5%. For ^212^Pb, the background recovery was significantly worse and there was a large variation between the imaging protocols and phantom setups (RC_BG_: 0.46 up to 0.92).

**Fig. 8:**
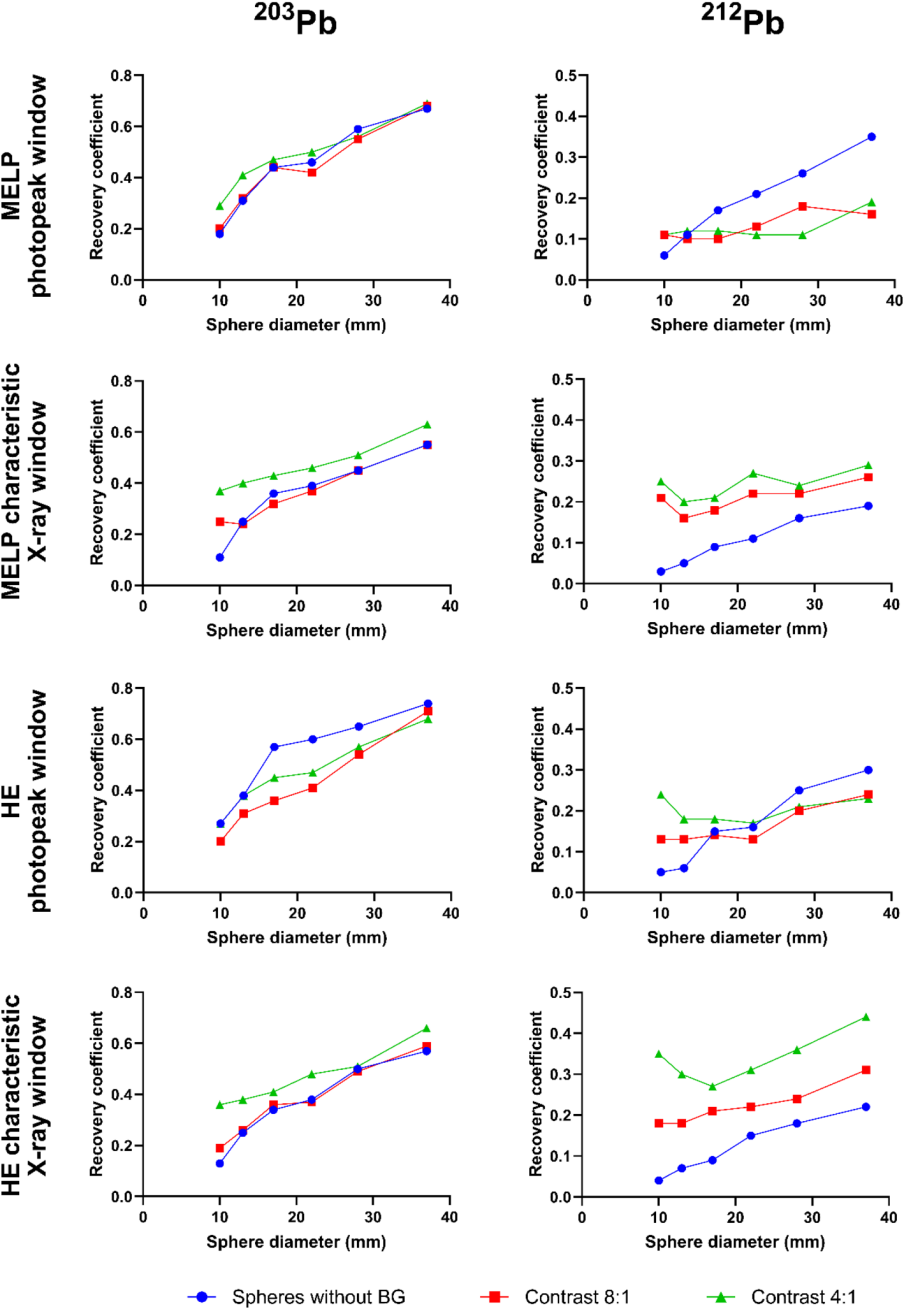
Recovery coefficients as a function of sphere diameter for ^203^Pb (left column) and ^212^Pb (right column) determined using the NEMA image quality phantom in different phantom setups (spheres without background activity, sphere-to-background activity concentration ratios of 8:1 and 4:1) acquired with MELP and HE collimators and different energy windows (photopeak window: 279 keV (^203^Pb) & 239 keV (^212^Pb); characteristic X-ray window: 72 keV (^203^Pb) & 79 keV (^212^Pb))

The recovery curves for all NEMA IQ phantom setups measured with different acquisition protocols are shown in Fig. 8. For ^203^Pb, the RC decreased continuously with decreasing sphere diameter. The RC curves for the measurement with MELP collimators and 279 keV window were the most similar between the different phantom setups. In contrast, the RCs for the 72 keV window increased for the 8:1 and 4:1 contrast setups compared to the setup with activity in the spheres only. For the ^212^Pb measurements without background activity, the 239 keV window images showed higher RCs than the 79 keV window images. In contrast, for the measurements with background activity, the 239 keV window images showed lower RCs than the 79 keV window images. In addition, the RCs of the three smallest spheres were similar or increased for the measurements with background activity.

**Table 5:** Calibration factors determined measuring a uniform cylindrical phantom filled with ^203^Pb and ^212^Pb

Nuclide	^203^Pb				^212^Pb			
**Collimator**	MELP		HE		MELP		HE	
**Energy window**	279 keV	72 keV	279 keV	72 keV	239 keV	79 keV	239 keV	79 keV
**Calibration factor (cps/MBq)**	124.9	507.8	82.5	367.8	131.7	603.5	119.1	367.0

## Discussion

Knowledge of the imaging properties of theragnostic radionuclides is necessary to enable adequate imaging and its application in terms of personalized radionuclide therapy. This is particularly important when introducing new radiopharmaceuticals to investigate pharmacokinetics, estimate therapeutic doses, and assess clinical feasibility. The scintigraphic imaging properties of ^203^Pb have not been previously studied, and for ^212^Pb, there are two studies investigating quantitative SPECT/CT imaging and acquisition and reconstruction parameters [36, 43]. However, fundamental imaging characteristics related to count rate performance and planar imaging were not considered. In our study, we focused on the scintigraphic imaging performance of ^203^Pb and ^212^Pb using clinically geared phantoms, considering planar and SPECT/CT imaging and the influence of dead time effects, collimator type, and tumor-to-background ratio.

### Sensitivity and count rate performance

The measured sensitivities for ^212^Pb were significantly higher than for ^203^Pb, although the gamma emission probabilities and the emission probabilities of the characteristic X-rays of ^212^Pb are lower than those of ^203^Pb. This is due to the occurrence of collimator-produced lead X-rays as well as septal penetration and down-scatter of high-energy photons from ^212^Pb progeny [43]. The MELP collimators showed a higher sensitivity for both isotopes, which is due to the smaller collimator thickness and the resulting larger acceptance angle (MELP: 4.15° vs. HE: 3.85°). In addition, the lower septal thickness and the resulting higher amount of septal penetration, especially for ^212^Pb, contribute to the higher sensitivity of the MELP collimators. The count rate performance for ^203^Pb showed an almost linear detector response for activities up to approximately 900 MBq where the count loss was less than 10%. The range of activity used clinically for ^203^Pb diagnostic imaging is between 200 MBq and 500 MBq [30, 44], so there is no need for a dead time correction. The progressive count rate loss for ^212^Pb with increasing activity is caused by extensive dead time effects that were evident in the measured energy spectra in terms of pulse pile-up, peak broadening and peak shifting. These extensive deadtime effects are induced by the presence of collimator-produced lead X-rays and septal penetration and down-scatter of high-energy photons from ^212^Pb daughter nuclides (e.g., 2.6 MeV from ^208^Tl) that are detected over the entire energy range of the detector. A count rate loss of 20% occurred even at very low activities of about 20 MBq for the MELP collimators and about 40 MBq for the HE collimators. This is two orders of magnitude lower than reported activities for other clinically used radionuclides such as technetium-99m (^99m^Tc) or lutetium-177 (^177^Lu), where a 20% count loss was observed only for activities above 1 GBq [45, 46]. There is a dose-escalation trial for [^212^Pb]Pb-DOTAMTATE where activities up to 296 MBq per cycle have been administered [27] and in a current dose-escalation study for [^212^Pb]Pb-VMT-α-NET [47] activities up to 370 MBq are to be used. It is therefore essential to correct for dead time related count losses in post-therapeutic ^212^Pb imaging, especially in quantitative SPECT. The activities where the maximum count rate occurs according to the PDM have been calculated, since in patient imaging it is not possible to determine whether the measured count rate is above or below the maximum count rate. In our study, these activities for ^212^Pb were 102 MBq and 89 MBq for the MELP collimators and 193 MBq and 166 MBq for the HE collimators. This could be a limitation for imaging at early time points as the activities at the maximum count rate according to the PDM may be exceeded. Measurements with the MELP collimators result in higher dead time effects compared to the HE collimators. This is due to the higher sensitivity of the MELP collimators and a higher incidence of septal penetration. Therefore, the use of HE collimators is more appropriate for clinical imaging with ^212^Pb, as higher dead time effects were found for the MELP collimators, making image quantification more difficult at higher activities. Higher count rate losses were observed for the measurements using the characteristic X-ray energy window compared to the photopeak window. This is most probably caused by the narrower energy window and therefore more pronounced dead time losses due to pulse pile-up effects. A relationship between dead time and energy window width has been reported by Silosky et al. [48] and Heemskerk et al. [49] for ^99m^Tc, and Ulribe et al. [50] reported also higher dead time effects for the lower energy photopeak window in ^177^Lu quantitative imaging. In our study we focused on imaging using the ^212^Pb photopeak at 239 keV and the characteristic X-rays. Further studies could investigate the ^208^Tl photopeaks at 511 keV and 583 keV, which appear to be less influenced by down-scatter. However, in the 511 keV peak only a small percentage is from primary photons and most counts are from pair production caused by the 2.6 MeV photons from ^208^Tl [43].

### Planar imaging of ^203^Pb and ^212^Pb

The evaluation of the planar images showed a better visual image quality of ^203^Pb compared to ^212^Pb regarding contrast, image and background noise, and sphere detectability (Fig. 4). In the images with activity in the spheres only, spheres with diameters ≥ 13 mm and ≥ 28 mm were visible for ^203^Pb and ^212^Pb, respectively. This suggests that lesions of this size with high tumor-to-background contrast are detectable in planar patient scans. For measurements with 8:1 and 4:1 sphere-to-background activity concentration ratios, only the largest spheres were visible for ^203^Pb and for ^212^Pb none of the spheres were detectable. This implies that planar imaging of smaller lesions with lower tumor-to-background contrast is difficult with ^203^Pb and may not be possible with ^212^Pb. The ^203^Pb images acquired with the characteristic X-ray window showed more artificial counts in the activity free lung insert compared to the images acquired with the 279 keV photopeak window. This is due to the fact that not only the characteristic X-rays of ^203^Pb are detected, but also the characteristic X-rays produced in the collimator, which have a loss of spatial information and result in increased counts in compartments of no activity [51]. All ^212^Pb images showed a high image and background noise which is due to the high amount of septal penetration and down-scatter of high-energy photons from ^212^Pb progeny. Another factor degrading the image quality of ^212^Pb could be image distortion due to pulse pile-up effects [52], which can occur at the activity levels used for phantom measurements with background activity (Table 2), as discussed above. Also noticeable in the 8:1 and 4:1 contrast images with ^212^Pb was the visibility of the PMT outlines. This is caused by the dead time related peak shift and the resulting apparent mistuning as the measured photopeak does not coincide with the energy window photopeak [53, 54]. In whole-body imaging, the PMT artifacts may not be visible, but can still cause degraded image quality [55].

### SPECT/CT imaging of ^203^Pb

All imaging protocols demonstrated the ability of SPECT/CT imaging with ^203^Pb. However, there were differences in imaging performance between the collimators and energy windows used. The images obtained with the MELP collimators showed slightly better image characteristics in terms of spatial resolution and image noise compared to the HE collimators. The worse resolution is a consequence of the larger hole diameter and the larger source to detector (crystal surface) distance due to the thicker HE collimator and the higher image noise is due to the lower sensitivity of the HE collimator [56, 57]. It was found that the images acquired with the 72 keV energy window had worse imaging properties regarding CRC, spatial resolution and count error in the lung insert compared to the 279 keV window images. The worse spatial resolution is a result of the lower gamma energy, which leads to a decrease in spatial resolution and a noticeably higher blurring [58]. The higher lung count error is due to the detection of collimator-produced lead X-rays in the 72 keV window, as described above. This can cause quantitative imaging and the resulting dosimetry to overestimate activity and dose in areas of no activity. The detectability of the spheres in the reconstructed images were similar for the four imaging protocols. In the images with activity only in the spheres, all spheres (d ≥ 10 mm) were visible, and in the images with background activity, spheres ≥ 13 mm were detectable (Fig. 6). Therefore, good lesion detectability is expected for clinical imaging with ^203^Pb at both very high and low tumor-to-background ratios. The measured calibration factors for ^203^Pb were higher for the MELP collimators than for the HE collimators due to the higher sensitivity of the MELP collimators, as described above. Additionally, the CFs for the 72 keV window were higher compared to the 279 keV window which is due to collimator produced lead X-rays that are detected in addition to the ^203^Pb X-rays and due to the higher detection efficiency at lower energies [51, 59]. For all imaging protocols and phantom setups, the background RCs showed only small deviations (≤ 5%) from the true activity concentrations suggesting a high level of quantification accuracy for regions of larger volume. The measured sphere RCs decreased continuously with decreasing sphere diameter due to the partial volume effect. This should be considered for dosimetry to avoid the activity and thus the calculated absorbed dose being underestimated for small objects. The RCs for the images acquired with MELP collimators in the 279 keV window showed the smallest variation between the different phantom setups. Therefore, good lesion quantification accuracy is expected when performing recovery curve based partial volume correction, even with varying tumor-to-background contrast. However, in clinical imaging, there are other factors influencing activity recovery, such as irregularly shaped lesions, lesion position, inhomogeneous activity distribution, and patient thickness, which may cause uncertainty in quantification accuracy [46, 60]. The quantification accuracy and the application of RCs to different phantoms (e.g. anthropomorphic phantoms) should be investigated in further research. Furthermore, the influence of different reconstruction parameters on image quality and quantification accuracy should be investigated.

### SPECT/CT imaging of ^212^Pb

The imaging protocols investigated for ^212^Pb showed a large variation in terms of quantitative imaging performance and visual image quality. It was found that the images acquired with the 79 keV energy window showed better performance regarding image noise and CNR compared to the 239 keV window. This is due to the higher count rates measured in the 79 keV window (Table 5). Although the planar sensitivities did not show large differences between the two energy windows, the count rates determined from the tomographic images were significantly higher for the 79 keV window due to the scatter correction applied and the elimination of the high fraction of scattered photons present in the 239 keV window (Fig. 3b). A high lung count error of more than 70% was observed for all imaging protocols due to a high septal penetration caused by high-energy photons from ^212^Pb daughter nuclides. This can lead to a significant overestimation of activity in volumes with low or no activity. Regarding visual image quality, spheres ≥ 13 mm were visible in the images without background activity. The images with background activity showed degraded image quality and sphere detectability was challenging (Fig. 7). Additionally, there is a high image noise resulting in a distortion of the spheres as they merge with the noisy background [61]. Images obtained with the HE collimators in the 79 keV window showed the best visual quality, as spheres ≥ 17 mm and ≥ 28 mm were distinguishable for the 8:1 and 4:1 images, respectively. This is consistent with the study by Kvassheim et al. [36], who also reported that HE 79 keV was the visually best protocol. In contrast to our study, SPECT/CT imaging was only investigated for NEMA phantom measurements without background activity. Based on our results, moderate lesion detectability is expected for clinical imaging with ^212^Pb at high tumor-to-background ratios, but is challenging at low tumor-to-background ratios. The measured CFs for ^212^Pb were similar to the work of Kvassheim et al. [36], who found that CFs were stable when activities above 1 MBq were acquired. The determined background recovery coefficients were lower for the 4:1 contrast images compared to the 8:1 contrast images and showed a significant underestimation of the activity concentration. Based on our count rate performance results, this is due to dead time related count losses and the higher activity in the FOV for the 4:1 contrast measurements. The dead time effects that occur could also cause the degraded image quality due to image distortion [52, 62]. The measured sphere RCs showed large variations between the different phantom setups, which makes recovery correction difficult and can result in quantification uncertainties. However, the sphere RCs may also be affected by count losses and should be determined in further studies using dead time corrected SPECT data. Therefore, SPECT/CT acquisitions of a NEMA phantom at different activity levels and the investigation of the count rate performance according to the PDM using the reconstructed SPECT data should be performed. In addition to dead time effects, the high proportion of patient scatter, collimator scatter and septal penetration of high-energy photons from ^212^Pb progeny can have a significant impact on quantification. Further studies could therefore investigate optimized reconstruction algorithms using improved corrections such as Monte Carlo based scatter corrections or resolution modeling [63, 64].

### Clinical application of ^203^Pb and ^212^Pb as theragnostic isotopes

The different imaging properties of ^203^Pb and ^212^Pb influence the possibility of clinical application as theragnostic isotopes in TAT. Based on our results and recent first-in-human imaging studies, post-treatment imaging with ^212^Pb is clinically feasible. However, the imaging characteristics of ^212^Pb showed a moderate image quality, as lesion detection is not reliable depending on the tumor-to-background ratio and a limited quantification accuracy is expected, making dosimetry challenging. Assuming that a valid quantification is possible, the qualitative imaging results of our study suggest that dose calculation could be applicable for larger lesions with high tumor-to-background ratios, but dosimetry may not be possible for small lesions. Tumor dosimetry is useful to assess a dose-response relationship, but dosimetry for organs at risk is important, especially for the kidney which is considered the dose-limiting organ in most peptide-based radionuclide therapies [65]. Dosimetry for the kidney and other larger normal tissues may be possible with ^212^Pb. However, the accuracy of the determination of pharmacokinetics, which is a prerequisite for dosimetry, may be limited because early imaging time points may not be quantifiable due to high dead time effects. Due to the short half-life of ^212^Pb, imaging at later time points results in low count statistics, which can degrade image quality [29] and affect quantification and dosimetry accuracy. Therefore, appropriate imaging time points for dosimetry with ^212^Pb need to be investigated. A method that may improve the count statistics for ^212^Pb SPECT/CT was presented by Griffiths et al. [66], in which the reconstructed images from the photopeak window and the characteristic X-ray window were summed.

Compared to ^212^Pb, ^203^Pb demonstrated superior imaging properties, making it an ideal diagnostic imaging surrogate. Due to the good visual image quality, image quantification and the longer half-life of ^203^Pb, it is well suited to investigate biodistribution and pharmacokinetics and to perform predictive dosimetry for ^212^Pb based radiopharmaceutical therapies. Dos Santos et al. [30] have already investigated dosimetry estimation for ^203/212^Pb-CA012 using planar whole-body ^203^Pb imaging. Our results showed that SPECT/CT imaging with ^203^Pb is definitely feasible and is expected to provide improved dosimetry accuracy compared to planar-based dosimetry. Diagnostic imaging with ^203^Pb can be used to identify patients who may benefit from a ^212^Pb TAT and for patient-specific dosimetry. Due to the elementally identical isotopes used, accurate prediction of biodistribution and pharmacokinetics is expected. Therefore, the ^203/212^Pb theragnostic pair has the potential to provide for a personalized treatment planning approach that considers absorbed doses to tumors and organs at risk, which may improve the efficacy of TAT. However, further research is needed on the applicability of ^212^Pb dead time correction, quantification accuracy and a dosimetry comparison of ^203^Pb and ^212^Pb based on phantom measurements and then on patient data.

## Conclusion

To the best of our knowledge, this is the first study investigating the scintigraphic imaging properties and acquisition protocols of ^203^Pb and comparing imaging performance with ^212^Pb. Our measurements demonstrated that planar and SPECT/CT imaging of ^203^Pb is possible with both MELP and HE collimators, with the MELP collimators showing slightly better imaging properties. Good visual image quality and quantitative imaging make ^203^Pb an ideal imaging surrogate to investigate pharmacokinetics and perform predictive dosimetry of ^212^Pb-labelled radiopharmaceuticals. In comparison, direct imaging of ^212^Pb was shown to be feasible, but less favorable due to high dead time effects even at low activities, which make image quantification and post-treatment dosimetry challenging. Therefore, further research regarding the applicability of ^212^Pb dead time correction and a dosimetry comparison of ^203^Pb and ^212^Pb based on phantom measurements and patient images is required. In conclusion, our results and first-in-human imaging studies revealed that SPECT/CT imaging with the ^203/212^Pb theragnostic pair is clinically feasible (using routine protocols for patients) and has great potential to make an important contribution to the progress of personalized targeted alpha therapy.

## Data Availability

The datasets used and/or analyzed during the current study are available from the corresponding author on reasonable request.

## Abbreviations

TAT: Targeted alpha therapy
LET: Linear energy transfer
RBE: Relative biological effectiveness
MELP: Medium-energy low-penetration
HE: High energy
FOV: Field of view
PDM: Paralyzable detector model
IQ: Image quality
OSEM: Ordered Subset Expectation Maximization
VOI: Volume of interest
CRC: Contrast recovery coefficient
CNR: Contrast-to-noise ratio
FWHM: Full width at half maximum
CF: Calibration factor
RC: Recovery coefficient
PMT: Photomultiplier tube
Cps: Counts per second
